# Relationship between long-term blood pressure fluctuations and visual field progression in primary open-angle glaucoma: a prospective cohort study

**DOI:** 10.1186/s40662-026-00490-4

**Published:** 2026-05-21

**Authors:** De-Fu Chen, Tao Wang, Jinrui Zhang, Chenmin Wang, Xiaonan Lu, Jingyu Xiao, Qiangjie Huang, Yuanbo Liang

**Affiliations:** 1 National Clinical Research Center for Ocular Diseases, Eye Hospital, Wenzhou Medical University, No. 270, Xue Yuan Xi Road, Wenzhou, 325000 China; 2 Glaucoma Research Institute, Wenzhou Medical University, Wenzhou, Zhejiang China

**Keywords:** Primary open-angle glaucoma, Blood pressure variability, Visual field progression, Risk factors

## Abstract

**Background:**

This study aimed to evaluate the association between long-term blood pressure (BP) variability (BPVR) and visual field (VF) progression in patients with primary open-angle glaucoma (POAG).

**Methods:**

In this prospective cohort study, linear mixed-effect models were used to assess the associations between BP metrics and VF progression rates. BPVR and intraocular pressure (IOP) variability during the follow-up period were calculated by dividing the respective standard deviation values by the mean values. Correlated non-fluctuating BP metrics (baseline, mean, maximum, and minimum BP) were combined using principal component analysis separately for systolic BP (SBP) and diastolic BP (DBP). The first principal component was included as a covariate. Interactions between covariates and time from baseline were modelled to evaluate their effects on VF progression rates.

**Results:**

A total of 157 eyes of 157 patients with POAG (mean baseline age, 56.3 ± 13.6 years) were included. VF progression was observed in 71 eyes (45%). These eyes showed a significantly higher incidence of disc haemorrhage (DH) than eyes without VF progression (36.6% vs. 15.1%, *P* = 0.002). In Cox proportional-hazard analysis, the presence of DH (hazard ratio [HR]: 2.60, 95% confidence interval [CI]: 1.58–4.28, *P* < 0.001) and higher systolic BPVR (SBPVR; HR: 1.12, 95% CI 1.00 to 1.24, *P* = 0.040) were significant predictors of VF progression. Additionally, SBPVR was significantly associated with faster VF progression (coefficient: − 0.127 dB/year per 1% increase, 95% CI − 0.247 to − 0.008, *P* = 0.037), whereas IOP variability (*P* = 0.284) and diastolic BPVR (DBPVR; *P* = 0.859) were not significantly associated with the progression rates.

**Conclusions:**

Long-term BP variability, particularly SBPVR, may be an independent risk factor for accelerated VF progression in POAG. Monitoring BP variability may help identify patients at higher risk of disease progression.

**Supplementary Information:**

The online version contains supplementary material available at 10.1186/s40662-026-00490-4.

## Background

Intraocular pressure (IOP) is a well-established risk factor for the progression of glaucoma [1–3], a neurodegenerative disease primarily affecting retinal ganglion cells [4]. Although IOP reduction is the primary therapeutic target for glaucoma, it is not the only factor influencing disease progression. A growing body of evidence suggests that vascular factors also contribute to both the pathogenesis and progression of glaucoma [5–7].

Numerous studies have indicated that blood pressure (BP) plays an important role in the development of primary open-angle glaucoma (POAG) [8, 9], particularly in patients with normal-tension glaucoma [10, 11]. However, the exact role of BP remains controversial, since previous studies have been inconsistent regarding which BP components most influence progression. For instance, while low diastolic BP and extreme nocturnal dipping have been identified as risk factors in several populations [12–14], other evidence points to elevated systolic BP and excessive morning BP surges as significant predictors of functional decline [12, 15]. Moreover, BP fluctuates over time, and BP variability (BPVR) or temporal changes in BP may be associated with visual field (VF) deterioration during the course of POAG [16, 17]. Nevertheless, isolating BPVR as an independent factor is challenging because it is closely correlated with other BP-related metrics, such as baseline BP and mean BP.

Therefore, we hypothesised that BPVR contributes to longitudinal changes in the VF in patients with POAG. This prospective study aimed to investigate whether long-term BPVR, assessed as visit-to-visit variability, is independently associated with glaucomatous VF changes and the rate of VF progression in POAG.

## Methods

### Study participants and procedures

This prospective cohort study included patients with POAG who were followed up and treated in the Wenzhou Glaucoma Progression Study (WGPS) between August 15, 2014, and April 16, 2024. The study was conducted in accordance with the Declaration of Helsinki and was approved by the Ethics Committee of the Eye Hospital of Wenzhou Medical University (No. KYK-2013-12). Before their inclusion in the study, the study participants received a full explanation of the study protocol and provided informed consent. The design of the WGPS has been described in detail in previous studies [18, 19]. For the present exploratory analysis, participants with POAG from the WGPS cohort were included if they had at least five reliable VF test results and a minimum follow-up period of two years. POAG was defined as characteristic glaucomatous optic nerve damage with corresponding VF defects, open anterior chamber angles on gonioscopy, and absence of secondary causes of glaucoma. Patients with other ocular or systemic conditions that could affect the optic nerve or visual function were excluded. A reliable VF test result was defined on the basis of the following previously established criteria [20]: the number of false-positive results of the test should be ≤ 15%, and that of fixation losses ≤ 33%, with no limit for false-negative VF tests. VF progression was evaluated according to Early Manifest Glaucoma Trial (EMGT) criteria [21]. Specifically, progression was defined as a significant deterioration in VF sensitivity at three or more identical test points in comparison with baseline, detected in three consecutive reliable VF examinations using Guided Progression Analysis (GPA) in the FORUM Glaucoma Workplace (software version 4.2, Carl Zeiss Meditec AG, Jena, Germany).

Only one eye per patient was included in the analysis. For patients with bilateral POAG, the eye demonstrating VF progression was selected when progression was unilateral. If both eyes were eligible for inclusion, one eye was randomly chosen.

### Clinical evaluations

VF examinations using standard automated perimetry (Humphrey Field Analyser 750i; Zeiss Corporation, Germany) were conducted at 3- to 6-month intervals. All tests were performed with the central 24-2 protocol on the basis of the Swedish Interactive Threshold Algorithm Standard [22]. IOP measurements at each visit were obtained using Goldmann applanation tonometry (GAP) (HAAG-STREIT 900 CM; Haag-Streit, Kloniz, Switzerland).

BP was measured at each visit by using an upper-arm digital sphygmomanometer (Hem-8102A; OMRON, Dalian, China). The participants were seated in a quiet environment for at least 5 min before the measurement. Two consecutive seated BP measurements were then obtained from both arms. Measurements were obtained during routine daytime clinic visits, typically between 8:00 a.m. and 5:00 p.m. To evaluate the multifaceted profile of BP, we categorised BP metrics into two distinct groups:

- BP-level metrics: Baseline BP was defined as the average of the readings obtained at the initial visit. For the longitudinal data, we calculated the mean BP (average of all readings throughout the follow-up period) as well as the maximum and minimum BP (the highest and lowest recorded values during the entire follow-up period), which represented the cumulative exposure and extreme pressure points, respectively.
- BPVR: To assess dynamic fluctuations of BP over time, we quantified systolic and diastolic BPVR (SBPVR and DBPVR, respectively) using the coefficient of variation (CV). The CV was calculated as (standard deviation [SD]/mean BP) × 100%, where SD was the SD of all BP measurements during follow-up. This normalised metric allowed the assessment of long-term variability independent of the mean BP level.

For baseline assessments, missing values were imputed using measurements obtained at the 3- or 6-month follow-up visit.

### Statistical analyses

Statistical analyses and data visualisation were performed using the free and open-source software R (version 4.1.3; R Project for Statistical Computing, Vienna, Austria). Categorical variables were expressed as counts and percentage (n, %), while continuous variables were expressed as mean ± SD. Demographic and clinical characteristics were compared between the groups. Normally distributed continuous variables were analysed using the Student’s *t*-test, whereas non-normally distributed variables were compared using the Mann–Whitney U test. Categorical variables were analysed using the chi-squared test.

Univariable and multivariable Cox proportional-hazard models were used to identify the potential risk factors for glaucoma progression. IOP-related parameters, including mean IOP and IOP variability during follow-up, were included as covariates in the regression analyses to account for their potential influence on glaucoma progression. Variables considered potentially associated with glaucoma progression based on prior literature and clinical relevance were first examined in univariable analyses. Variables with *P* < 0.20 were subsequently entered into multivariable models using a backward stepwise selection procedure based on the minimum Akaike information criteria.

In addition, univariable and multivariable linear mixed-effect models (LMMs) with random intercepts and slopes were used to estimate disease progression rates and evaluate associations with the clinical parameters of interest. In these models, the mean deviation (MD) value at each visit was used as the outcome variable. Eye identification number and follow-up time were included as random intercept and slope terms, respectively. Baseline MD was included as a fixed-effect covariate to account for differences in baseline disease severity across the eyes. To evaluate the potential multicollinearity and redundancy between the baseline MD and the random intercept, variance inflation factors (VIFs) were calculated. A VIF of 1.038 confirmed that the inclusion of baseline MD as a fixed effect did not introduce significant bias or compromise the stability of the estimated progression rates. Factors with *P* < 0.20 in univariable analyses were further incorporated into the multivariable models.

To address the inherent collinearity among multiple BP metrics (baseline, mean, maximum, and minimum BP), we applied principal component analysis (PCA). The primary objective of this approach was to consolidate these highly correlated parameters into a single, comprehensive “composite BP magnitude index,” thereby statistically isolating the overall pressure burden from the dynamic dimension of visit-to-visit variability. PCA was conducted separately for systolic BP (SBP) and diastolic BP (DBP) by using patient-level summary measures. Scree plots were used to visualise the proportion of explained variance and guide component selection (Supplementary Figure S2). The first principal component (PC1) accounted for 83.6% of the variance in SBP and 78.4% of that in DBP. In this context, PC1 can be clinically interpreted as the “weighted average” or “sustained pressure baseline” of a patient throughout the follow-up period. The loading structure revealed that the four input metrics contributed almost equally to the components (loading magnitudes: 0.48 to 0.53). This balanced loading pattern confirmed that the first principal components of SBP and DBP (SBPPC1 and DBPPC1, respectively) can serve as robust composite representations of the overall sustained BP. Using this approach, we effectively captured the shared variance of these collinear parameters, ensuring that our model could distinguish between the cumulative pressure magnitude (represented by PC1) and the temporal pressure instability (quantified by SBPVR and DBPVR).

## Results

A total of 157 eyes of 157 patients with POAG (mean baseline age, 56.3 ± 13.6 years; see Table 1) were included in the analysis. Of these, 72 patients (45.9%) were female. The mean MD at baseline was − 7.13 ± 5.19 dB. Over a mean follow-up period of 6.1 ± 2.4 years, participants underwent an average of 16.9 ± 7.4 visits. Furthermore, 64 patients (40.8%) reported a history of systemic arterial hypertension; 36 (22.9%) reported a diagnosis of diabetes mellitus during the interviews; and all were receiving systemic treatment. At the latest follow-up, 71 patients (45%) showed VF progression.

**Table 1:** Demographic and ocular characteristics in patients with primary open-angle glaucoma in the progression and non-progression groups

Characteristic	Overall (N = 157)	Progression (n = 71)	Non-progression (n = 86)	*P* value
Age at baseline (years)	56.3 ± 13.6	58.4 ± 13.0	54.5 ± 13.9	**0.038**
Sex, female	72 (45.9)	39 (54.9)	33 (38.4)	**0.038**
Baseline MD (dB)	− 7.13 ± 5.19	− 6.60 ± 4.90	− 7.56 ± 5.40	0.321
Mean IOP (mmHg)	14.30 ± 1.90	14.41 ± 1.91	14.21 ± 1.89	0.514
IOPVR (%)	13.2 ± 3.5	13.4 ± 3.6	13.0 ± 3.4	0.594
Diabetes, n (%)	36 (22.9)	20 (28.2)	16 (18.6)	0.156
Hypertension, n (%)	64 (40.8)	31 (43.7)	33 (38.4)	0.502
Disc haemorrhage, n (%)	39 (24.8)	26 (36.6)	13 (15.1)	**0.002**
SBPVR (%)	7.6 ± 2.3	7.8 ± 2.1	7.3 ± 2.4	0.193
SBPPC1	0.00 ± 1.83	− 0.11 ± 1.76	0.09 ± 1.89	0.737
DBPVR (%)	9.5 ± 2.5	9.9 ± 2.3	9.3 ± 2.7	**0.118**
DBPPC1	0.00 ± 1.77	0.18 ± 1.63	− 0.15 ± 1.88	0.255
Migraine	10 (6.4)	6 (8.5)	4 (4.7)	0.332

*DBP* = diastolic blood pressure; *DBPVR* = diastolic blood pressure variability; *IOP* = intraocular pressure; *IOPVR* = intraocular pressure variability; *MD* = mean deviation; *PC1* = principal component 1; *SBP* = systolic blood pressure; *SBPVR* = systolic blood pressure variability. Significant *P* values are shown in bold

### VF progression based on the guided progression analysis

In a comparison of demographic characteristics between patients showing VF progression with those with stable VF, the patients showing progression had older average age (mean baseline age: 58.4 ± 13.0 years vs. 54.5 ± 13.9 years, *P* = 0.038) and included a greater proportion of females (54.9% vs. 38.4%, *P* = 0.038). In addition, 26 patients (36.6%) in the progression group had a history of disc haemorrhage (DH), which was significantly higher than the corresponding number and proportion in the stable VF group (13 patients [15.1%], *P* = 0.002). The baseline clinical characteristics were otherwise similar between the groups (MD: − 6.60 ± 4.90 dB vs. − 7.56 ± 5.40 dB, *P* = 0.321; mean IOP: 14.41 ± 1.91 mmHg vs. 14.21 ± 1.89 mmHg, *P* = 0.514). In addition, the two groups showed no significant differences in the non-fluctuating BP parameters represented by the SBPPC1 (− 0.11 ± 1.76 vs. 0.09 ± 1.89, *P* = 0.737) and DBPPC1 (0.18 ± 1.63 vs. − 0.15 ± 1.88, *P* = 0.255) or long-term BP fluctuation measures (SBPVR: 7.8% ± 2.1% vs. 7.3% ± 2.4%, *P* = 0.193; DBPVR: 9.9% ± 2.3% vs. 9.3% ± 2.7%, *P* = 0.118).

The risk factors for VF progression, defined as a binary outcome on the basis of the Early Manifest Glaucoma Trial criteria, are summarised in Tables 2 and 3. In the univariate proportional-hazard model, the presence of DH was a significant predictor of VF progression (hazard ratio [HR]: 2.52, 95% confidence interval [CI]: 1.54–4.13, *P* < 0.001), as was increased SBPVR (HR: 1.10, 95% CI 1.00 to 1.21, *P* = 0.063), although the latter was only near statistical significance. In the multivariable analysis, the results were similar, confirming that DH (HR: 2.60, 95% CI 1.58 to 4.28, *P* < 0.001) and increased SBPVR (HR: 1.12, 95% CI 1.00 to 1.24, *P* = 0.040) were both correlated significantly with VF progression.

**Table 2:** Univariable Cox proportional-hazard analysis of risk factors for visual field progression in patients with primary open-angle glaucoma

	Univariate model		
Characteristic	Hazard ratio	95% confidence interval	*P* value
Age at baseline (years)	1.01	0.99 to 1.03	0.396
Sex, female	1.39	0.87 to 2.22	**0.165**
Baseline MD (dB)	1.00	0.95 to 1.05	0.93
Mean IOP (mmHg)	1.01	0.89 to 1.14	0.891
IOPVR (%)	1.02	0.95 to 1.10	0.513
Diabetes	1.07	0.64 to 1.80	0.791
Hypertension	1.03	0.64 to 1.65	0.898
Current smoking	1.16	0.60 to 2.27	0.658
History of alcohol consumption	0.78	0.49 to 1.25	0.302
Disc haemorrhage	2.52	1.54 to 4.13	** < 0.001**
History of migraine	0.84	0.36 to 1.95	0.679
SBPVR (%)	1.10	1.00 to 1.21	**0.063**
SBPPC1	0.96	0.85 to .09	0.509
DBPVR (%)	1.04	0.95 to 1.14	0.348
DBPPC1	1.03	0.90 to 1.17	0.683

*DBP* = diastolic blood pressure; *DBPVR* = diastolic blood pressure variability; *IOP *= intraocular pressure; *IOPVR* = intraocular pressure variability; *MD* = mean deviation; *PC1* = principal component 1; *SBP* = systolic blood pressure; *SBPVR* = systolic blood pressure variability. Significant *P* values are shown in bold

**Table 3:** Multivariable Cox proportional-hazards analysis of risk factors for visual field progression in patients with primary open-angle glaucoma

	Multivariable model		
Characteristic	Hazard ratio	95% confidence interval	*P* value
Sex, female	1.42	0.88 to 2.30	0.150
Disc haemorrhage	2.60	1.58 to 4.28	** < 0.001**
SBPVR (%)	1.12	1.00 to 1.24	**0.040**

*SBPVR* = systolic blood pressure variability. Significant *P* values are shown in bold

### Rate of MD in VF progression

**Fig. 1:**
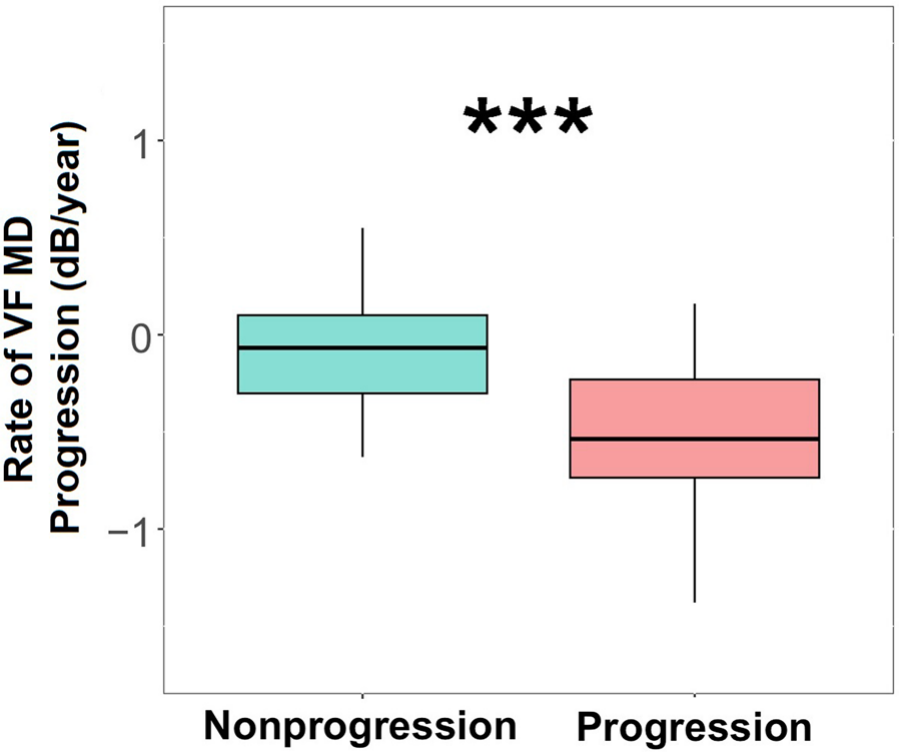
Rate of progression of the mean deviation (MD) of the visual field (VF) in the non-progression and progression groups

The mean rate of change in the MD of VF was − 0.29 dB/year (interquartile range [IQR], − 0.57 to − 0.02) over a mean follow-up period of 6.2 years (IQR, 4.2–8.6). Figure 1 shows that the mean rate of change of the MD in the VF progression cohort was − 0.53 dB/year (IQR, − 0.74 to − 0.23 dB/year), while the mean progression in MD/year in the stable VF cohort was − 0.09 dB/year (IQR, − 0.31 to 0.11 dB/year), and the difference was again statistically significant (*P* < 0.001).

Univariable and multivariable LMMs were used to evaluate the factors associated with the rate of VF change over time (Tables 4 and 5). In the univariable analysis, a higher (less negative) baseline MD of VF was significantly associated with the rate of change in the MD over time (coefficient: 0.944 dB/year per 1-dB increase in baseline MD; 95% CI 0.897 to 0.991, *P* < 0.001). In addition, greater long-term SBPVR was significantly associated with the rate of MD progression (coefficient: − 0.150 dB/year per 1% increase; 95% CI − 0.257 to − 0.042; *P* = 0.007). In contrast, DBPVR was not significantly associated with the rate of MD decline (*P* = 0.163).

**Table 4:** Univariable linear mixed-effect models evaluating factors associated with the rate of change in the mean deviation of the visual field over time in patients with primary open-angle glaucoma

	Univariate model		
Characteristic	Coefficient (dB/year)	95% confidence interval (dB/year)	*P* value
Age at baseline (years)	0.001	− 0.017 to 0.019	0.900
Sex, female	− 0.352	− 0.838 to 0.133	**0.154**
Baseline MD (dB)	0.944	0.897 to 0.991	** < 0.001**
Mean IOP (mmHg)	0.087	− 0.042 to 0.216	**0.187**
IOPVR (%)	2.679	− 4.358 to 9.714	0.453
Diabetes	0.112	− 0.475 to 0.699	0.708
Hypertension	0.009	− 0.494 to 0.511	0.972
Current smoking	− 0.290	− 0.988 to 0.408	0.413
History of alcohol consumption	− 0.076	− 0.580 to 0.429	0.768
Disc haemorrhage	0.107	− 0.453 to 0.667	0.707
History of migraine	0.707	− 0.246 to 1.660	**0.145**
SBPVR (%)	− 0.150	− 0.257 to − 0.042	**0.007**
SBPPC1	− 0.031	− 0.174 to 0.113	0.676
DBPVR (%)	− 0.069	− 0.167 to 0.028	**0.163**
DBPPC1	− 0.045	− 0.187 to 0.097	0.531

Estimates are intended for a 1-unit increase unless specified otherwise. *DBP* = diastolic blood pressure; *DBPVR* = diastolic blood pressure variability; *IOP* = intraocular pressure; *IOPVR* = intraocular pressure variability; MD = mean deviation; *PC1* = principal component 1; *SBP* = systolic blood pressure; *SBPVR* = systolic blood pressure variability. Significant *P* values are shown in bold

**Table 5:** Multivariable linear mixed-effect models evaluating factors associated with the rate of change in the mean deviation of the visual field over time in patients with primary open-angle glaucoma

	Multivariable model		
Characteristic	Coefficient (dB/year)	95% confidence interval (dB/year)	*P* value
Sex, female	− 0.250	− 0.738 to 0.238	0.313
Baseline MD (dB)	0.934	0.887 to 0.981	** < 0.001**
Mean IOP (mmHg)	0.070	− 0.058 to 0.198	0.284
History of migraine	0.530	− 0.421 to 1.482	0.272
SBPVR (%)	− 0.127	− 0.247 to − 0.008	**0.037**
DBPVR (%)	− 0.010	− 0.115 to 0.010	0.859

Estimates are intended for a 1-unit increase unless specified otherwise. *DBPVR* = diastolic blood pressure variability; *IOP* = intraocular pressure; *MD* = mean deviation; *SBPVR* = systolic blood pressure variability. Significant *P* values are shown in bold

Multivariable analysis yielded similar results. A higher (less negative) baseline VF MD (coefficient: 0.934 dB/year per 1-dB increase in baseline MD, 95% CI 0.887 to 0.981, *P* < 0.001) and greater long-term variability in SBP (coefficient: − 0.127 dB/year per 1% increase, 95% CI − 0.247 to − 0.008, *P* = 0.037) remained significantly associated with a faster rate of VF progression. However, the parameters that correlated with non-fluctuating BP, such as SBPPC1 (*P* = 0.676) and DBPPC1 (*P* = 0.531), were not significantly correlated with the rate of change in MD in the univariate analysis.

## Discussion

In this cohort investigation, we examined the relationship of BPVR and non-fluctuating BP parameters with VF progression in patients with POAG. In summary, increased BPVR, especially SBPVR, was associated with both VF progression and faster rates of VF decline.

A growing body of evidence has shown that systemic BP plays an important role in the development and progression of POAG, although the findings across studies remain inconsistent [23–25]. Many BP-related parameters are closely interrelated and can introduce multicollinearity into regression analyses. In this study, PCA was applied to the correlated BP metrics to generate orthogonal components that captured the overall BP level while minimising collinearity. BPVR is inherently related to mean BP, making it difficult to isolate their independent effects on VF progression. The use of the CV, calculated as the SD divided by the mean BP, and expressed as a percentage, partially mitigated this issue by normalising the variability relative to the average BP levels.

Pham et al. [26] reported that a higher mean DBP is associated with increased rates of loss in the MD of VF. Other studies have shown that lower systolic perfusion pressures and DBP are associated with open-angle glaucoma [12]. Our previous study demonstrated the relationship between lower DBP and structural progression [14]. However, the relationship between BP and glaucoma is complex. For example, the Barbados Eye Studies reported that a lower SBP was associated with a greater risk of glaucoma progression [8].

Interestingly, in our models, a higher mean IOP showed a non-significant trend toward slower VF progression. This finding may reflect reverse causation or treatment-related confounding, whereby eyes perceived to be at a higher risk of progression or demonstrating faster progression may have received more intensive IOP-lowering treatment during follow-up, resulting in a lower observed mean IOP.

The Thessaloniki Eye Study demonstrated that iatrogenic reductions in DBP to levels below 90 mmHg were associated with increased optic disc cupping and reduced neuroretinal rim area in comparison with spontaneously occurring DBP values below 90 mmHg, suggesting that treatment-related BP reductions may contribute to structural changes in the optic nerve [27]. Several studies have also suggested that excessive antihypertensive treatment leads to substantial BP reductions, and the consequent decrease in ocular perfusion pressure may contribute more to glaucoma progression than hypertension itself [25, 28]. These findings highlight the importance of careful monitoring of systemic BP during long-term management of patients with glaucoma.

Previous studies have reported mixed findings regarding the relationship between systemic BP and glaucoma progression. Importantly, the average BP level and BPVR represent two distinct dimensions of systemic haemodynamic regulation. While average BP reflects the overall level of systemic perfusion pressure, visit-to-visit BPVR reflects the stability of systemic perfusion over time. In the present study, PCA was used to capture long-term BP levels, whereas BPVR was analysed separately. Many previous studies have quantified BPVR using the SD of BP measurements. For example, Lee et al. [17] reported that patients with normal-tension glaucoma showing VF progression exhibited greater long-term BP fluctuations over a 7-year follow-up period in comparison with those who did not show progression. Similarly, increased DBPVR has been associated with a greater risk of MD deterioration [26].

In our study, greater long-term SBPVR was associated with both VF progression and faster rates of VF decline, whereas DBPVR was not significantly associated with these outcomes. Similar findings were reported by Lee et al. [17], who observed that SBPVR was significantly correlated with the rate of VF progression (*P* = 0.007), whereas the association with DBPVR was weaker (*P* = 0.048). In addition, a large population-based study demonstrated that increased long-term SBPVR was associated with a significantly higher risk of developing POAG (*P* < 0.001) [16]. One possible explanation is that large fluctuations in systemic BP may impair endothelial function and disrupt vascular autoregulation, potentially through alterations in nitric oxide metabolism or damage to the neurovascular unit [29, 30]. Moreover, visit-to-visit variability in SBP has been associated with factors related to arterial stiffness, including age, smoking status, and vascular health [31]. In the present study, SBPVR showed consistent associations with both the occurrence of progression in the GPA analysis and faster rates of VF decline in the LMMs, further supporting its potential role as a risk factor for POAG progression.

This study had some limitations that require consideration. First, the present analysis focused on long-term visit-to-visit BPVR, although short-term BP fluctuations, such as nocturnal hypotension or marked nocturnal drops in diastolic BP or mean arterial pressure, have also been associated with glaucoma risk [32–34]. These factors were not directly evaluated in the present study and may have influenced systemic BPVR. Since BP measurements were obtained during routine clinical visits rather than through ambulatory or 24—h monitoring, these short-term variations could not be directly assessed. Second, certain antihypertensive medications may exert protective effects against glaucoma [25, 35, 36]. However, due to the limited information on specific medication classes during earlier follow-up periods, we were unable to assess their potential neuroprotective effects or their possible influence on BPVR. In addition, topical antiglaucoma medications such as β-blockers may have systemic effects that could influence BP. In our cohort, many participants were untreated at baseline, and IOP-lowering therapy was typically initiated after VF progression was confirmed during follow-up. However, the potential confounding effects of specific medication classes were not formally evaluated in the present analysis. Furthermore, although baseline MD was included as a covariate to adjust for initial disease severity, a VIF of 1.038 confirmed that this inclusion did not introduce significant multicollinearity or bias in the estimation of progression rates. Finally, the generalisability of these findings should be interpreted with caution, since this was a single-centre study conducted in a Chinese population with a relatively young mean age, and most participants had early to moderate glaucoma based on MD values.

## Conclusion

In conclusion, this prospective longitudinal cohort study indicated that increased variability in BP, particularly in SBP, may be a risk factor for glaucomatous changes in POAG. This study demonstrated the importance of measuring the long-term variability in POAG treatment.

## Supplementary Information

Additional file 1 (DOCX 195 KB)

## Data Availability

No datasets were generated or analysed during the current study.

## Abbreviations

BP: Blood pressure
BPVR: Blood pressure variability
CV: Coefficient of variation
DBP: Diastolic blood pressure
DBPPC1: First principal component of diastolic blood pressure
DBPVR: Diastolic blood pressure variability
DH: Disc haemorrhage
GPA: Guided progression analysis
IOP: Intraocular pressure
LMMs: Linear mixed-effect models
MD: Mean deviation
PC1: First principal component
PCA: Principal component analysis
POAG: Primary open-angle glaucoma
SBP: Systolic blood pressure
SBPPC1: First principal component of systolic blood pressure
SBPVR: Systolic blood pressure variability
SD: Standard deviation
VF: Visual field
VIF: Variance inflation factor
WGPS: Wenzhou glaucoma progression study
