# Supplementary material for: Relationship between long-term blood pressure fluctuations and visual field progression in primary open-angle glaucoma: a prospective cohort study
Source: Eye Vis (Lond). 2026 May 21;13:21. doi: 10.1186/s40662-026-00490-4 (PMC13192074; doi:10.1186/s40662-026-00490-4)

**Supplementary Materials**

**Supplementary Figure S1. Representative case of high systolic blood pressure variability and visual field (VF) progression during follow-up.** **a** Longitudinal systolic blood pressure (SBP) measurements obtained during routine clinic visits, showing marked visit-to-visit variability. **b** Corresponding trajectory of visual field mean deviation (MD) over time, demonstrating a progressive decline in the visual field.


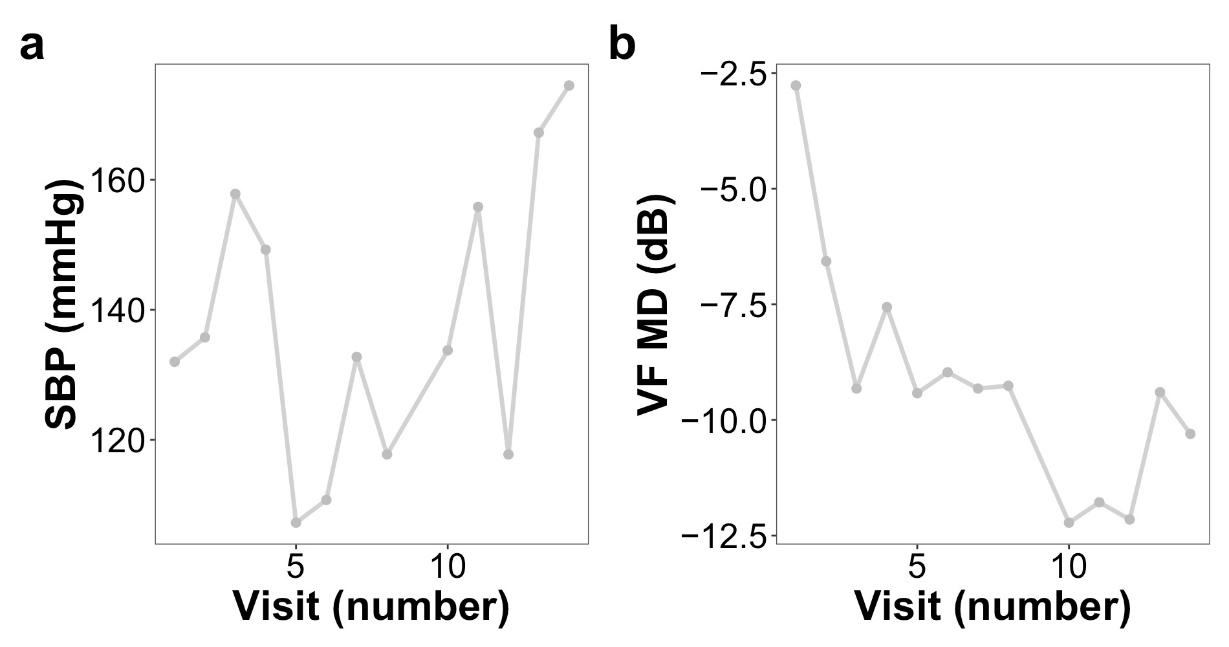


**Supplementary Figure S2. Scree plots illustrating the variance explained by the principal components for blood pressure parameters. a** Scree plot of the principal components for systolic blood pressure (SBP). **b** Scree plot of the principal components for diastolic blood pressure (DBP). The plots show the proportion of variance accounted for by each principal component derived from four BP metrics (baseline, mean, maximum, and minimum BP). For both SBP (**a**) and DBP (**b**), the first principal component (PC1) captured the vast majority of the total variance (83.6% and 78.4%, respectively), which was characterised by a distinct “elbow” at the first component. This high explanatory power justifies the use of PC1 as a robust composite representation of the overall blood pressure magnitude, effectively consolidating collinear parameters into a single dimension.


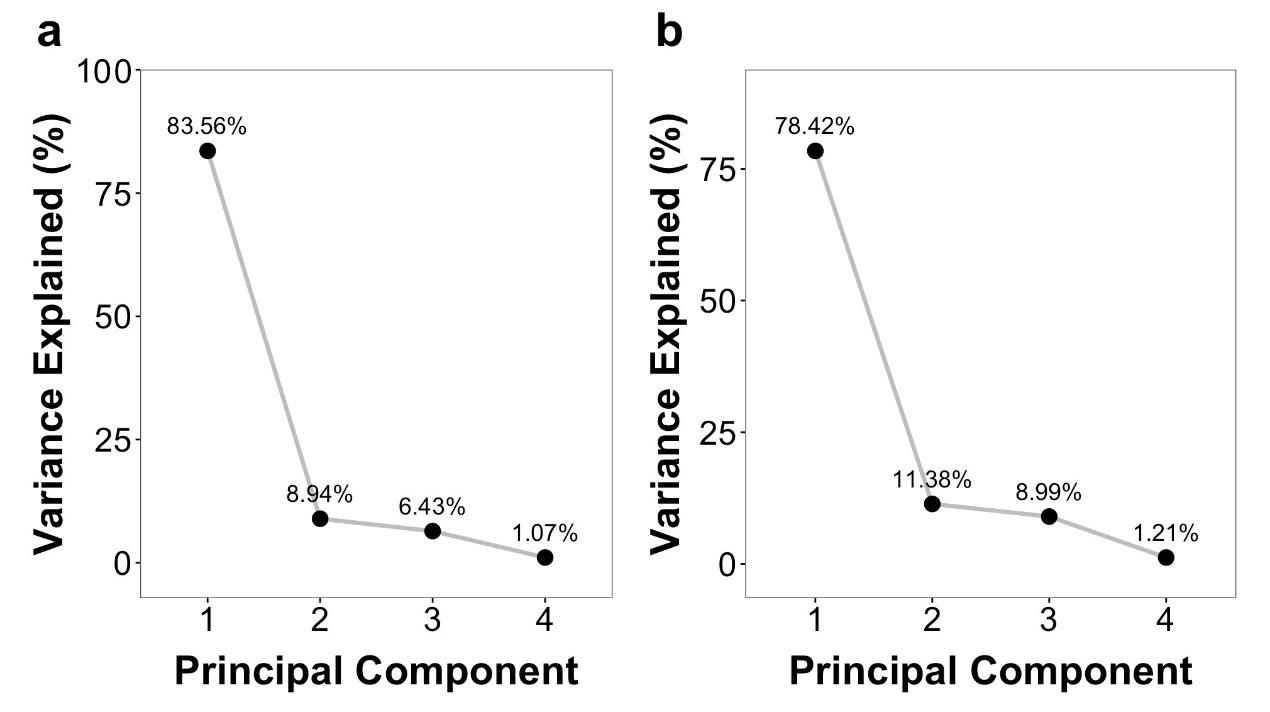

Supplement: Supplementary file 1 — Additional file 1 (DOCX 195 KB) [file 40662_2026_490_MOESM1_ESM.docx]
